# Toward artificial intelligence in dental prosthesis planning — a preliminary in-silico feasibility study

**DOI:** 10.1186/s12903-025-06778-6

**Published:** 2025-08-31

**Authors:** Michael del Hougne, Philipp del Hougne, Isabella Di Lorenzo, Christian Höhne, Johannes Schrenker, Marc Schmitter

**Affiliations:** 1 Department of Prosthodontics, University of Würzburg, Pleicherwall 2, Würzburg, 97070 Germany; 2 Univ Rennes, CNRS, IETR - UMR 6164, Rennes, F-35000 France

**Keywords:** Artificial neural network, In-silico data set, Prosthodontic workflow

## Abstract

**Background:**

Dental prosthesis planning is a multi-faceted and nuanced process of conceiving individual treatment plans based on dental findings and in line with established treatment guidelines. The aim of this study was to assess whether an artificial neural network (ANN) provided with sufficient training data could approximate this process.

**Methods:**

Dental prosthesis planning was abstracted as a mapping from dental findings to choices of dental prosthesis. The problem was framed as a multi-output multi-class classification. An ANN was trained via supervised learning to approximate dental prosthesis planning based on synthetic datasets of dental findings and corresponding prosthesis choices. The accuracy on unseen test data was examined as a function of the ANN’s random initializations, the training set sizes, and the ANN architecture.

**Results:**

Within the scope and limitations of this study, the ANN achieved an accuracy of 99.51% (± 0.15).

**Conclusions:**

The ability of ANNs to learn dental prosthesis planning was confirmed within the limitations of this preliminary in-silico study. The findings of this study corroborate that ANNs have the potential to support clinicians by providing automated recommendations for choices of dental prosthesis consistent with relevant rules, ultimately supporting and enhancing clinicians’ decision making. Moreover, such ANNs may, in principle, enable advanced patient self-assessment of treatment needs and improve patient care in prosthodontics.

**Supplementary Information:**

The online version contains supplementary material available at 10.1186/s12903-025-06778-6.

## Introduction

Dental prosthesis planning is a complex process in which practitioners engage multiple times per working day. Given a set of dental findings, several treatment possibilities need to be evaluated, e.g., to replace insufficient restorations or to provide a new dental prosthesis as a treatment for missing teeth. Treatments with fixed dental prosthesis (FDP), removable dental prosthesis (RDP), and complete dentures (CD), among others, can be involved. The German healthcare system has the special feature that patients are either publicly or privately insured. For publicly insured patients, a standard-care treatment option can be determined based on a specific set of rather complex rules. Based on the widespread deployment of artificial intelligence (AI) in digital medicine, AI assistance in proposing a choice of dental prosthesis that follows the applicable set of rules could represent a workload relief for practitioners. It could furthermore enable advanced patient self-assessment of treatment needs.

Modern AI tools are based on artificial neural networks (ANNs). Their architectures are originally inspired by neurobiology [1, 2]. Trained ANNs can approximate complex input-to-output mappings through supervised learning, using representative training examples to implicitly learn the desired function without needing it to be explicitly defined. While the universal approximation theorem suggests that sufficiently large artificial neural networks (ANNs) can, in principle, approximate any continuous function on a bounded domain, it does not guarantee that such functions are learnable from finite or practical amounts of data [3]. AI tools have emerged across all areas of digital medicine and profoundly impact medical practitioners [4, 5]. Within the realm of dentistry, AI tools are mainly developed to assist with the analysis of X-ray images [6]. Approaches have been made to the detection and localization of caries and hypomineralization [7], while others have explored the use of AI in the field of dental implants [8, 9]. Within prosthodontics, artificial intelligence has shown promise as a tool for automated tooth reconstruction and various dental applications, but further research is needed to compare different AI approaches and evaluate their clinical performance [10, 11]. As ANNs can accurately learn and approximate complex mappings, progress in automated dental prosthesis planning could be made by training ANNs that adhere to standard-care treatment rules or authoritative opinions of universities or experienced practitioners.

To the authors’ knowledge, no significant progress has been made in AI assisted choices of dental prosthesis. The present preliminary study addressed this research gap and specifically aimed to establish the feasibility of AI dental prosthesis planning and to gain insights into its requirements in terms of AI architecture and training. By defining dental prosthesis planning as a mapping from a set of dental findings to a set of choices of dental prosthesis, the aim of this study was to train an ANN to be capable of performing dental prosthesis planning and evaluating varying data set sizes and varying ANN architectures upon their impact on accuracy. To conduct this preliminary study under well-controlled conditions, a synthetic data set of pairs of dental findings and corresponding choices of dental prosthesis was generated.

The null hypothesis was that the ANN cannot perform, plan, indicate, or choose a dental prosthesis, similar to a human dentist or when trained on unknown dental data.

## Methods

The study focused on one jaw, mapping 16 dental findings to 16 associated choices of dental prosthesis, in line with the World Dental Federation (FDI) scheme featuring 16 teeth in the upper or lower jaw. For simplicity, there were 10 possible dental findings and 4 possible choices of dental prosthesis, as summarized in Table 1. The utilized abbreviations were based on the German scheme for treatment plans [12, 13]. The dental findings were interpreted as an overall evaluation (i.e. sufficient, insufficient, condemned, preservation worthy) of each item. Normally, more comprehensive data sets are required with restorative, periodontal, endodontic, and orthodontic findings to conclude each overall evaluation. Especially when evaluating the choice of dental bridge or RDP, the periodontal status of abutment teeth is critical. For simplicity, the present study did not incorporate such detailed data sets, and implants were excluded as they are not part of the German standard-care treatment options.

**Fig. 1:**
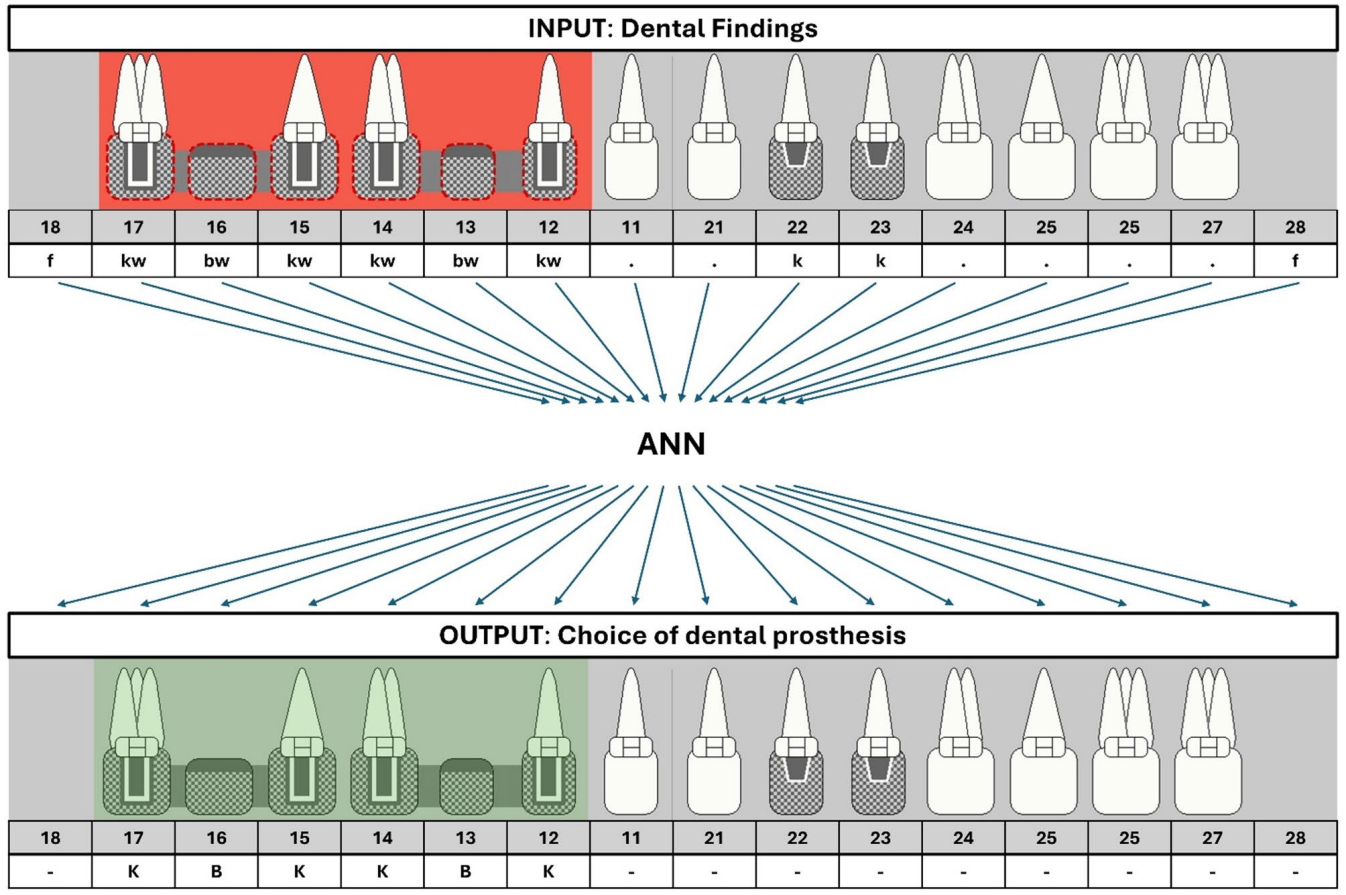
Systematic illustration of principle of AI dental prosthesis planning. ANN mapping exemplary input (set of 16 dental findings) to output (set of 16 choices of dental prosthesis). Insufficient FDPs are marked with red and corresponding choices of dental prosthesis with green. Symbols of dental findings and choice of dental prosthesis visualize the abbreviations detailed below each symbol; these abbreviations are defined in Table 1

The dental prosthesis planning was formalized into a mapping from one 16-element categorical input vector to another 16-element categorical output vector, as illustrated in Fig. 1. The attribute “categorical” highlights the limited number of discrete categories (as opposed to continuous values) that each vector entry could take. Thereby, the dental prosthesis planning was framed as a multi-output multi-class classification problem which was a very compact formulation, in comparison to AI tools developed for other problems like X-ray image analysis which take large matrices with continuous input values as input.

The study was conducted by a multidisciplinary team. The code for data synthesis and the ANN was developed and coded by a senior physician with six years of experience, board-certified in prosthodontics, and a physicist with over six years of experience in coding, including diverse applications of artificial neural networks. The validity of the synthetic data sets was tested by two independent experienced dentists, as detailed below.

**Table 1:** Overview of utilized abbreviations

Dental finding		Choices of dental prosthesis	
f	missing tooth, not replaced by FDP or RDP	-	no dental prosthesis required
.	tooth, sufficient	K	crown
x	tooth to be extracted (condemned)	B	bridge unit
k	crown, sufficient (FDP)	E	tooth to be replaced with RDP
b	bridge unit, sufficient (FDP)		
e	replaced tooth with RDP, sufficient		
ww	insufficient natural tooth (e.g. carious lesion), preservation worthy		
kw	insufficient crown, renewal required (FDP)		
bw	insufficient bridge unit, renewal required (FDP)		
ew	insufficiently replaced tooth with RDP, renewal required		

### Synthetic generation of labelled data sets

Firstly, data sets were generated synthetically. A rule was implemented that required a single or a sequence of missing teeth treated with pontics (i.e. FDP) to be surrounded by crowned teeth on both sides, resulting in an absence of cantilever FDPs. If bridge units were marked insufficient, the abutments of the FDPs were marked insufficient as well, and vice versa. If an RDP was present in the dental findings, either all or none of the replaced teeth had to be insufficient and thus with indication for renewal. RDPs were present as complete dentures or partial clasp dentures. For the choice of dental prosthesis, a rule was implemented, stating that the wisdom teeth did not require a replacement with dental prosthesis, once missing. If the choice of dental prosthesis required teeth to be replaced by an RDP, in case of an existing one, it also had to be renewed. Furthermore, the necessity of replacing four or more missing teeth resulted in an RDP, in line with the German scheme for treatment plans. Lastly a rule was implemented that every second data set had to include an FDP within the overall set of dental prosthesis choices. This approach was necessary to ensure sufficient representation of FDPs, as they would otherwise have been rare under the standard rules for treatment planning.

A Python program (v. 3.12, Python Software Foundation) in Visual Studio Code (v. 1.95.3, Microsoft Corporation) was written for the generation of the random dental findings and corresponding choices of dental prosthesis according to these rules. In total, 20,000 pairs of random dental findings and corresponding choices of dental prosthesis were generated. It was verified that each of the 20,000 data sets was unique and not duplicated. The data sets were then split evenly, with 10,000 used for training and validation, and 10,000 reserved exclusively for testing the ANN’s performance on truly unseen data. This approach was employed to eliminate the risk of data leakage (by construction, none of the test examples were seen during training), and to ensure that the test results reflect the model’s generalization ability.

To test the validity of the synthetic data sets, two independent observers (experienced dentists – a senior physician, 6 years experienced, and a professor and head of prosthetic department, 20 + years experienced, both board certified prosthodontists - who were already familiar with German treatment plans) were calibrated by the initial explanation of the rules and the resulting choices of dental prosthesis. They were provided with an identical sample of 100 data sets that were randomly chosen from the 20,000 synthetically generated data sets. Both observers identified no mistakes in the logic of the dental findings and the resulting choices of dental prosthesis. As mentioned, of these 20,000 data sets, 10,000 data sets were used for testing to assess the ANN’s performance on unseen data. A subset of *N*_cal_ data sets drawn randomly from the other 10,000 data sets was used for training and validation, as detailed in the next section. Each training was repeated 10 times, each time based on a new independent draw of *N*_cal_ data sets from the 10,000 ones available for training and validation.

This in-silico study was conducted on a system with a Ryzen Z1 Extreme APU (Advanced Micro Devices, Inc., USA) equipped with 16 GB of 7500 MHz DDR5 RAM, running Windows 11 Home (version 23H2).

### Artificial neural network: Architectural design, training and testing

The baseline ANN was referred to as M0 in this study. All ANN models were implemented using Python (v. 3.12, Python Software Foundation) as the programming language, interpreter, and runtime environment, along with the following libraries and frameworks: TensorFlow (v. 2.18.0, tensorflow.org), Keras (v. 3.6.0, keras.io), and Scikit-learn (v. 1.5.2, scikit-learn.org). As a result, the ANNs were executed locally on the machine.

The basis for training M0 were paired sets of a 16-element categorical input vector (dental findings) and the corresponding 16-element categorical output vector (choices of dental prosthesis). The categorical input vector was converted into a numeric vector by assigning an index to each category, while the categorical output vector was transformed via one-hot encoding into a binary matrix representation. The input layer of M0 accepted a numeric 16-element vector corresponding to a set of dental findings. This input layer was followed by an embedding layer that transformed each entry of the input vector into a dense 64-element vector, outputting a sequence of 16 64-element vectors. This step was followed by a bidirectional Long Short-Term Memory (LSTM) layer with 128 units per direction, outputting a sequence of 16 256-element vectors, a dropout layer setting half of the entries to zero, a dense layer (with 64 units and Rectified Linear Unit (ReLU) activation) outputting a sequence of 16 64-element vectors, another dropout layer setting half of the entries to zero, and a final dense layer with 4 units and softmax activation outputting a sequence of 16 4-element vectors. Each 4-element vector was a probability distribution over the four possible categories in the choice of dental prosthesis. Finally, a categorical 16-element output vector was obtained by choosing the category with the largest probability for each tooth. To train the ANN, a standard categorical cross-entropy loss function was used which compared the model’s probability distribution over the possible categories in the 16 choices of dental prosthesis with the one-hot encoding of the ground-truth output vectors.

To train the ANN, a total of *N*_cal_$\:\le\:$ 10,000 data sets were randomly drawn from the available 10,000 data sets. 80% of these *N*_cal_ data sets were used for actual training, whereas the other 20% of the *N*_cal_ data sets were used for validation. The latter allowed monitoring the validation loss and applying early stopping of the training if no improvement was observed for 10 epochs, with the best model weights restored. Early stopping helped to prevent overfitting, as did the dropout layers in the ANN architecture. The ANN was trained with the Adam optimizer using an initial learning rate of 10^−3^ which was reduced by a factor of 0.5 if the validation loss plateaued for five consecutive epochs, until a minimum learning rate of 10^−7^ was reached. The training contained up to 200 epochs with a batch size of 32.

After training, the ANNs’ accuracies were evaluated on an unseen test data set including 10,000 examples of dental findings and corresponding choices of dental prosthesis.

**Fig. 2:**
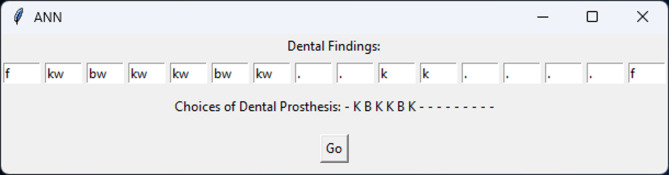
Graphical user interface (GUI). The exemplary findings and choices of dental prosthesis are identical to Fig. 1

A graphical user interface (GUI) was implemented for the proposed AI tool for dental prosthesis planning. The GUI enabled manually entering a set of dental findings and obtaining the corresponding set of choices of dental prosthesis proposed by the trained ANN. An example of the GUI is displayed in Fig. 2.

### Systematic analysis of ANN architecture and training requirements

A systematic analysis was conducted to evaluate how the trained model’s accuracy was affected by the ANN architecture and training conditions:

- The dynamics of training M0 with a training data set size (*N*_cal_) of 10, 100, 1,000, and 10,000 were evaluated.
- The influence of the training data set size was evaluated, by training M0 using 21 different values of *N*_cal_ between 50 and 7,000. For each value N_cal_, 10 different random initializations were conducted with a random selection of the fixed set of 10,000 training data sets each.
- Regarding the ANN architecture, M0 (detailed above) and five variations thereof (M1-M5) were tested, as summarized in Table 2. For M1, the LSTM layer of M0 was replaced with a dense layer of equal size. For M2, an additional dense layer with 32 units was added to the M0 architecture. For M3, the dense layer from M0 was removed. M4 contained a doubled number of units of all layers from M0. For M5, the number of units of all layers of M0 was halved. For analysis of the ANN architecture, M0, M1, M2, M3, M4 and M5 were trained with *N*_cal_=300 to be able to observe pronounced differences. For each architecture, 10 different random initializations were conducted with a random selection of 300 of the fixed set of 10,000 training data sets each.

**Table 2:** Details of the six considered model architectures. A comparison of their performances is displayed in Fig. 5

Name	Architecture	Description
M0	Embedding > LSTM (128) > Dropout (0.5) > Dense (64) > Dropout (0.5) > Dense (softmax)	N/A
M1	Embedding > Dense (128) > Dropout (0.5) > Dense (64) > Dropout (0.5) > Dense (softmax)	LSTM layer of M0 replaced with dense layer.
M2	Embedding > LSTM (128) > Dropout (0.5) > Dense (64) > Dropout (0.5) > Dense (32) > Dropout > Dense (softmax)	M0 with additional dense layer.
M3	Embedding > LSTM (128) > Dropout (0.5) > Dense (softmax)	M0 without its dense layer.
M4	Embedding > LSTM (256) > Dropout (0.5) > Dense (128) > Dropout (0.5) > Dense (softmax)	M0 with doubled units in each layer.
M5	Embedding > LSTM (64) > Dropout (0.5) > Dense (32) > Dropout (0.5) > Dense (softmax)	M0 with halved units in each layer.

### Statistical significance of accuracy differences

SPSS Statistics (Version 29, IBM) was utilized for statistical analysis. To compare multiple independent groups, the Kruskal-Wallis test was applied, followed by pairwise comparisons using the Dunn test with Bonferroni correction. Effect sizes were calculated for each comparison. The significance level was set at α = 0.05.

## Results

**Fig. 3:**
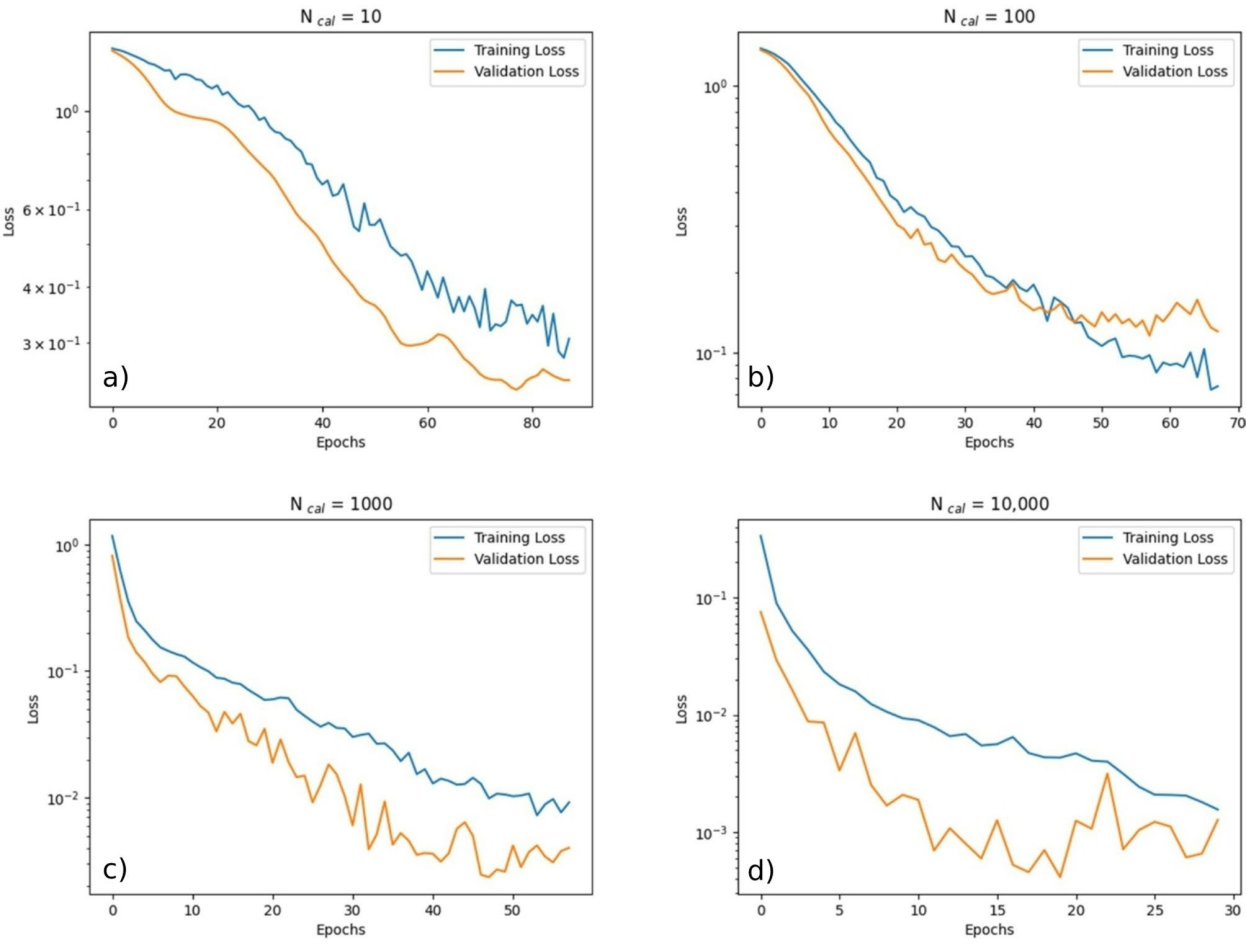
Exemplary plots of training loss and validation loss vs. training epochs for M0. The corresponding values of the number *N*_cla_ of data sets used for training (including validation) are 10 **a**, 100 **b**, 1000 **c** and 10,000 **d**, as indicated in the title of each subfigure

The dynamics of one training run with M0 for four different values of *N*_cal_: 10, 100, 1,000, and 10,000 were examined, as illustrated in Fig. 3. As expected, the training was stopped once the validation loss stopped decreasing. It was apparent that fewer epochs were needed for the training process to converge, if *N*_cal_ was larger. Moreover, it was apparent that lower values of the loss function were achieved, if *N*_cal_ was larger.

**Fig. 4:**
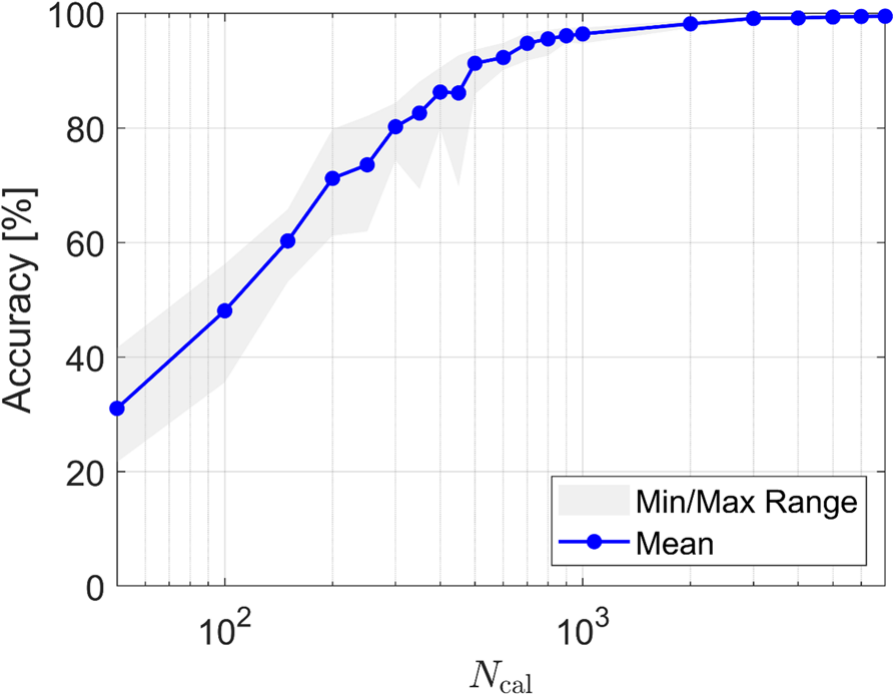
Influence of the number N_cal_ of data sets available for training (including validation) on the achieved accuracy of M0. The shaded grey area indicates the fluctuations across different training runs with different random initializations

The analysis of the size of *N*_cal_ on the final achieved accuracy was defined as the percentage of correct sets of choices of dental prosthesis. M0 was trained with 21 different values of *N*_cal_. For each value, the training was repeated 10 times with different random initializations, as displayed in Fig. 4 (corresponding data documented in Table S1). Two clear trends were observed: (i) The achieved accuracy increased as *N*_cal_ was increased, and (ii) its dependence on the random initialization decreased as *N*_cal_ was increased. With *N*_cal_ = 700, the accuracy was ∅94.78% (± 1.45). With the largest value of *N*_cal_ = 7,000, the accuracy was ∅99.51% (± 0.15). Therefore, the null hypothesis was confidently rejected. The ANN was clearly capable of correctly predicting choices of dental prosthesis for unseen dental findings. The improvements in accuracy as a function of *N*_cal_ were not linear and roughly exponential for low values of *N*_cal_. The marginal gains from increasing *N*_cal_ beyond 1,000 vanished for larger values of *N*_cal_.

**Fig. 5:**
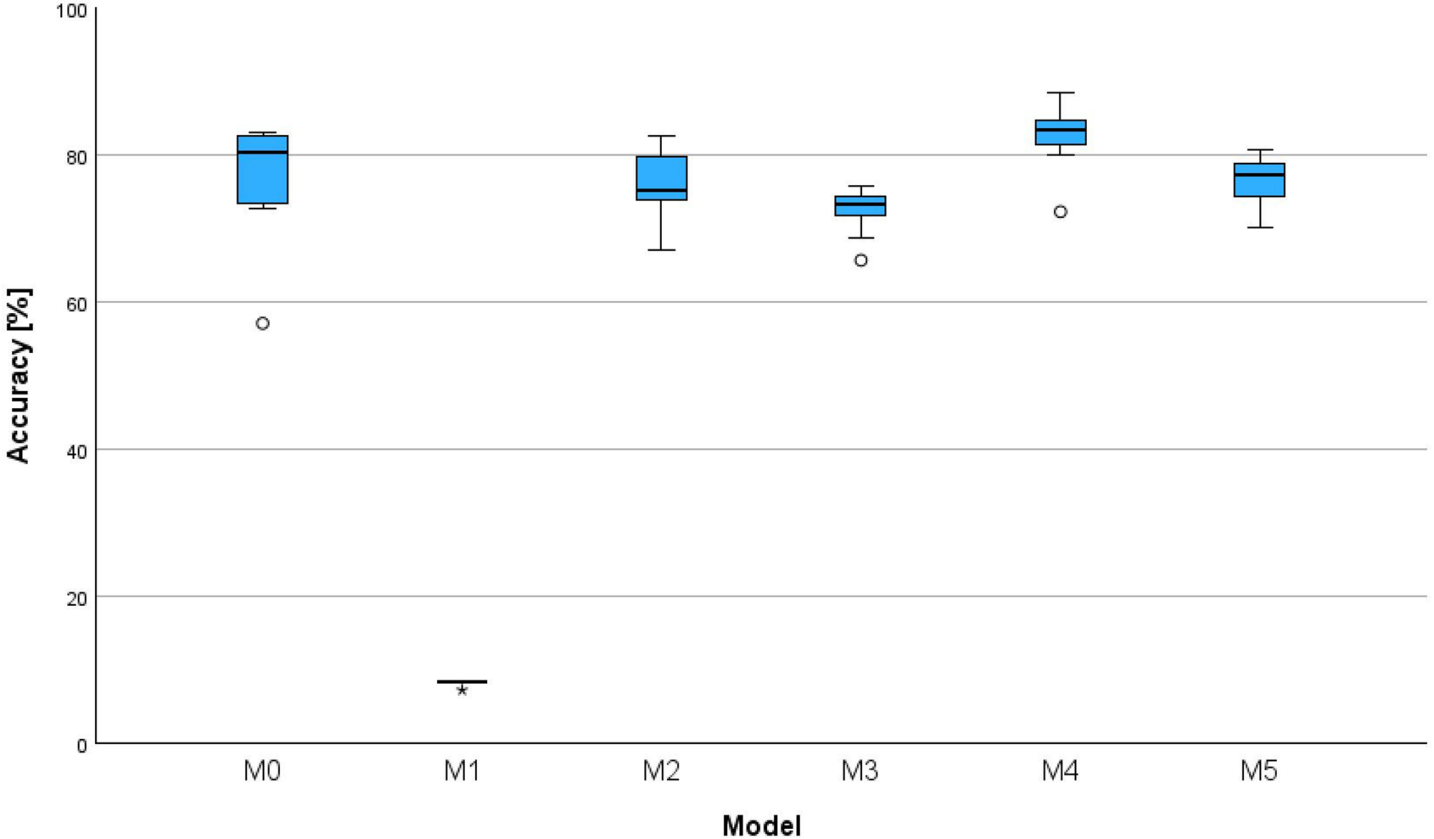
Boxplot of achieved accuracy in percentages for six model architectures (M0-M5). The considered model architectures (M0-M5) are detailed in Table 2

The analysis of the influence of the ANN’s architecture on its accuracy with a deliberately low value of *N*_cal_ = 300 revealed pronounced differences between the six models, as illustrated in the boxplots in Fig. 5 (corresponding data documented in Table S2).

M1 (∅8.18% (± 0.31), 95% CI [7.94, 8.41]) had the lowest accuracy. It performed significantly worse than M0 (∅77.33% (± 7.62), 95% CI [71.58, 83.97] (*p* < 0.001, *r* = 0.95), M2 (∅76.18% (± 4.65), 95% CI [72.76, 79.68]) (*p* = 0.007, *r* = 0.78), M4 (∅82.58% (± 4.06), 95% CI [79.52, 85.64]) (*p* < 0.001, *r* = 1.29) and M5 (∅76.59% (± 3.28), 95% CI [74.11, 79.06]) (*p* = 0.006, *r* = 0.79). It can be concluded that replacing the LSTM layer of M0 with a simpler dense layer resulted in a drastic loss of accuracy for M1. However, removing the dense layer from M0 only resulted in a marginally worse (*p* = 0.651, *r* = 0.45) accuracy for M3 (∅72.64% (± 3.01), 95% CI [70.37, 74.90]). Meanwhile, M4 with doubled number of units in each layer achieved a significantly higher accuracy than M3 (*p* = 0.006, *r* = 0.79). Overall, M4 achieved the highest accuracy and slightly outperformed M0. Details on statistical significance of the accuracy differences are summarized in Table 3.

**Table 3:** Statistical significance and effect size of accuracy differences between pairwise comparisons of the models (M0-M5)

Test pairs	Statistical significance	Effect size
M1-M3	0.398	0.50
M1-M2	0.007	0.78
M1-M5	0.006	0.79
M1-M0	< 0.001	0.95
M1-M4	< 0.001	1.29
M3-M2	1.000	0.29
M3-M5	1.000	0.29
M3-M0	0.651	0.45
M3-M4	0.006	0.79
M2-M5	1.000	0.00
M2-M0	1.000	0.16
M2-M4	0.360	0.50
M5-M0	1.000	0.16
M5-M4	0.378	0.50
M0-M4	1.000	0.34

The training process of M0 with *N*_cal_ = 7,000 followed by testing with 10,000 unseen data sets took approximately 10 min to complete with the hardware utilized in this study. Thus, training time and computing requirements were considered as practically feasible, especially if more powerful hardware is employed in the future.

## Discussion

### Model complexity

Increasing the complexity of the neural network model architecture led to improved accuracy under the specific conditions of this study. Generally, an ANN should be designed such that it achieves high accuracy while keeping its architecture as simple and streamlined as possible. This follows Occam’s Razor, the philosophical principle favoring simpler explanations. In the context of ANNs, this principle implies that effective models should avoid including parameters beyond what is minimally necessary to describe the systems they represent because simpler models enable improved generalization and are less prone to overfitting. Nevertheless, the quantitative performance details of the ANN architectures tested in this study cannot be generalized for other research questions, as they are tailored for this specific application.

This study highlights the practicality and feasibility (in terms of computing requirements, training time, required training examples) of developing and training a light-weight ANN for this task. At this in-silico stage, given all its limitations, an artificial neural network (ANN) is neither essential nor capable of achieving perfect performance. While rule-based approaches suffice for simple scenarios, more complex data sets involving multiple treatments or comprehensive data sets will require the flexibility and learning capacity of ANNs.

### Translational barriers

While the presented approach may, in principle, contribute to improving patient care, several translational barriers must be addressed before clinical implementation. These include the need for high-quality, annotated real-world data, compliance with data protection regulations and ethical standards, validation in diverse patient populations, and integration into existing clinical workflows [14, 15].

### Data generation and biases

As the methodology of this study involved the development of a code capable of generating large synthetic data sets, no methods for data augmentation such as flipping the dental findings and choices of dental prosthesis between quadrants were deployed. The methodology provided a controlled and consistent labeling process that eliminated challenges of inconsistent human annotations [16]. This approach enabled efficient scaling of the training data volume. Furthermore, the methodology ensured that the data set contained, as far as possible, a wide variety of dental findings and that the corresponding treatment plans were free of logical errors. Given the critical importance of data quality for training an ANN, the training data were independently reviewed and validated by two calibrated observers. However, the synthetic data sets did not fully reflect real-world scenarios, and the variability of the examples was not “biological”. For instance, canines often persist in a significantly reduced dentition, while other teeth might be lost earlier. Such patterns were not accounted for by the code for generating synthetic dental findings and, therefore, could not be learned by the ANNs.

Furthermore, the enforced inclusion of an FDP in every second data set introduced a distributional bias that may lead to overfitting to this artificial pattern. Consequently, the model could achieve high accuracy by exploiting the predictable alternation of FDP presence, rather than learning robust features for FDP detection. This may limit generalizability to naturally distributed data sets and result in misleading performance metrics during validation. The abbreviations of the dental findings generally represent the overall evaluation of each tooth and are intended to encompass all relevant aspects. In real life, these findings are typically recorded by dentists following a comprehensive clinical assessment of the patient. Although decision-making criteria are based on the local healthcare system and its specific requirements - such as tooth preservation worthiness - they are often influenced and biased by individual dentists’ perceptions and experiences. Therefore, training the ANN with comprehensive real-world data sets with authoritative treatment opinions from universities or experienced practitioners could provide valuable decision support for less experienced clinicians and contribute to more consistent treatment planning.

### Future directions

For future studies, data sets could be based on anonymized patient data to reflect realistic dental findings and prosthesis choices. Although the dental findings in this study included the overall evaluation of each tooth and aimed to encompass all relevant aspects, the use of comprehensive data sets—including restorative, periodontal, endodontic, and orthodontic findings, as well as the patient’s medical and dental history—would enhance clinical relevance. Implants were not considered, as they are not part of the German standard-care treatment options. Implant placement requires complex planning, particularly regarding available bone volume and the patient’s medical history, and such complexities were not captured in the training data used in this study. This represents a limitation of the present work. The treatment guidelines of the German healthcare system for publicly insured patients were selected, providing fixed and clear rules for all dental findings; however, these rules limit generalizability and represent a limitation. Moreover, the method in this study focused on a single jaw and could be extended to combined dental prosthesis planning for the upper and lower jaw. The absence of occlusal context represents a limitation of the present study. Overall, these enhancements would increase the complexity significantly and therefore probably substantially increase the required training data set size for clinically acceptable levels of accuracy.

Collecting patient data for a training data set size of 1000 already presents a technical hurdle while not ensuring an equal distribution of all choices of dental prosthesis, as an accumulation of certain types of prosthetic treatments driven by the characteristics of the patient population. One might be concerned that high amounts of patient data could be required to successfully train an ANN for complex real-world conditions. However, this burden could be alleviated by pre-training the ANN on synthetic data sets, as done here, and then fine-tuning the ANN with real patient data. Moreover, when utilizing anonymized patient data for training, data protection measures must be implemented, and ethical complications may arise. In this context, an advantage of this study’s approach would be the ANN’s local and offline training and execution, enabling controlled data protection.

### Safety and explainability

The rapid rise of AI in computing has sparked concern about its lack of understandable, explainable outputs—especially in biomedicine, where patient safety is critical [17]. To mitigate the risk of AI-driven overtreatment—a growing concern as algorithms increasingly influence clinical decisions—it is crucial to embed proactive safety mechanisms that prioritize harm avoidance over unchecked intervention [18]. Such frameworks aim to balance precision and caution, aligning computational decisions with ethical standards that protect patient welfare.

## Conclusion

To summarize, in this preliminary in-silico feasibility study, the ability of ANNs to learn dental prosthesis planning in a well-controlled environment based on synthetic generated data sets was explored. A set of dental plannings was mapped to a corresponding set of choices of dental prosthesis, following the specific set of complex rules for standard-care treatments in the German healthcare system. With sufficient training examples and a suitable ANN architecture, an accuracy of ∅99.51% (± 0.15) was achieved. Thereby, the main hypothesis that an ANN can accurately approximate the mapping from dental findings to choices of dental prosthesis was validated and the null hypothesis was convincingly rejected.

Altogether, deep learning in dentistry is expected to contribute to future dental and oral healthcare improvements [19]. The systematic analysis of the required ANN architecture and training data size for AI-based dental prosthesis planning in this preliminary study revealed valuable insights into minimal requirements. The outcomes of this study provide confidence that the presented AI-based method for dental prosthesis planning holds promise. Future work will build on the presented preliminary results obtained with synthetic data by applying the methodology established in this study to real patient data. Trained with comprehensive real-world data sets, the ANN-based approach presented in this study could develop real-life impact by enabling patient self-assessment of treatment needs, appointment prioritization, clinical auditing in public dental care, and dental insurance estimations.

## Supplementary Information

Supplementary Material 1.

## Data Availability

The data sets supporting the conclusions of this article are included within the article. The underlying code for this study and training/validation data sets are not publicly available for proprietary reasons.

## Abbreviations

ANN: Artificial neural network
AI: Artificial intelligence
*N*_cal_: Subset of data set
M: Model architecture
GUI: Graphical user interface
LSTM: Long Short-Term Memory
FDP: Fixed dental prosthesis
RDP: Removable dental prosthesis
