# Supplementary material for: Toward artificial intelligence in dental prosthesis planning — a preliminary in-silico feasibility study
Source: BMC Oral Health. 2025 Aug 31;25:1386. doi: 10.1186/s12903-025-06778-6 (PMC12398987; doi:10.1186/s12903-025-06778-6)
Supplement: Supplementary file 1 — Supplementary Material 1. [file 12903_2025_6778_MOESM1_ESM.docx]

Supplementary Tables

| N_cal_ | Trial 1 | Trial 2 | Trial 3 | Trial 4 | Trial 5 | Trial 6 | Trial 7 | Trial 8 | Trial 9 | Trial 10 | Mean | Std. dev. |
| --- | --- | --- | --- | --- | --- | --- | --- | --- | --- | --- | --- | --- |
| 50 | 41.62 | 38.91 | 30.97 | 37.33 | 21.69 | 33.56 | 23.14 | 28.62 | 28.07 | 26.60 | 31.05 | 6.36 |
| 100 | 48.00 | 42.36 | 56.25 | 52.44 | 51.79 | 51.32 | 56.00 | 43.65 | 35.62 | 43.51 | 48.09 | 6.33 |
| 150 | 62.73 | 53.16 | 58.56 | 60.20 | 64.46 | 61.06 | 60.82 | 65.80 | 59.84 | 56.25 | 60.29 | 3.53 |
| 200 | 77.93 | 62.61 | 79.79 | 75.71 | 61.62 | 74.03 | 73.25 | 61.22 | 72.80 | 73.12 | 71.21 | 6.50 |
| 250 | 75.30 | 65.54 | 82.08 | 61.96 | 75.83 | 73.04 | 79.81 | 72.16 | 75.17 | 74.87 | 73.58 | 5.70 |
| 300 | 82.92 | 78.33 | 80.20 | 74.37 | 84.39 | 82.05 | 80.04 | 82.91 | 75.33 | 81.73 | 80.23 | 3.15 |
| 350 | 85.67 | 82.12 | 69.36 | 82.03 | 83.29 | 84.99 | 86.45 | 84.18 | 79.91 | 88.07 | 82.61 | 4.96 |
| 400 | 87.60 | 87.09 | 79.99 | 82.24 | 88.69 | 86.25 | 90.56 | 86.55 | 86.82 | 86.99 | 86.28 | 2.89 |
| 450 | 85.88 | 89.67 | 90.73 | 86.60 | 86.11 | 69.86 | 85.02 | 92.28 | 92.69 | 82.34 | 86.12 | 6.29 |
| 500 | 90.43 | 90.60 | 93.23 | 93.50 | 92.68 | 91.58 | 93.67 | 89.32 | 85.93 | 91.96 | 91.29 | 2.25 |
| 600 | 92.83 | 90.16 | 90.85 | 93.55 | 94.85 | 93.55 | 93.42 | 91.11 | 91.10 | 91.66 | 92.31 | 1.45 |
| 700 | 95.81 | 96.23 | 93.20 | 94.96 | 95.38 | 94.35 | 93.62 | 96.69 | 91.81 | 95.78 | 94.78 | 1.45 |
| 800 | 96.65 | 92.64 | 96.20 | 94.90 | 96.17 | 96.71 | 95.92 | 94.15 | 97.11 | 95.13 | 95.56 | 1.30 |
| 900 | 96.50 | 96.99 | 97.52 | 96.65 | 97.21 | 95.28 | 95.89 | 94.92 | 94.89 | 95.22 | 96.11 | 0.94 |
| 1000 | 97.52 | 96.05 | 96.43 | 95.85 | 96.11 | 96.73 | 96.18 | 97.20 | 94.76 | 97.46 | 96.43 | 0.80 |
| 2000 | 98.38 | 97.94 | 97.51 | 97.46 | 97.79 | 98.33 | 98.81 | 98.77 | 98.17 | 98.77 | 98.19 | 0.48 |
| 3000 | 99.05 | 99.13 | 99.05 | 99.42 | 99.04 | 99.05 | 99.19 | 98.92 | 99.23 | 99.19 | 99.13 | 0.13 |
| 4000 | 99.13 | 99.37 | 98.94 | 99.37 | 99.26 | 98.87 | 99.40 | 99.15 | 99.32 | 99.25 | 99.21 | 0.17 |
| 5000 | 99.52 | 99.23 | 99.50 | 99.38 | 99.37 | 99.31 | 99.03 | 99.47 | 99.46 | 99.46 | 99.37 | 0.14 |
| 6000 | 99.44 | 99.55 | 99.30 | 99.57 | 99.35 | 99.49 | 99.55 | 99.58 | 99.30 | 99.55 | 99.47 | 0.11 |
| 7000 | 99.57 | 99.70 | 99.59 | 99.16 | 99.43 | 99.49 | 99.55 | 99.63 | 99.61 | 99.41 | 99.51 | 0.15 |

***Table S1: Raw data underlying Figure 4.*** *For each considered value of N*_cal_ *(first column), the accuracies of ten training runs of M0 are indicated, as well as their mean and standard deviation (std. dev.).*

| Model | Trial 1 | Trial 2 | Trial 3 | Trial 4 | Trial 5 | Trial 6 | Trial 7 | Trial 8 | Trial 9 | Trial 10 | Mean | Std. dev. |
| --- | --- | --- | --- | --- | --- | --- | --- | --- | --- | --- | --- | --- |
| M0 | 83.11 | 81.83 | 78.24 | 82.58 | 57.15 | 81.99 | 83.00 | 79.00 | 72.80 | 73.57 | 77.33 | 7.62 |
| M1 | 8.28 | 8.28 | 8.28 | 8.28 | 8.28 | 8.28 | 8.28 | 8.28 | 7.25 | 8.28 | 8.18 | 0.31 |
| M2 | 74.74 | 81.95 | 74.01 | 74.93 | 82.77 | 71.22 | 75.43 | 67.22 | 79.70 | 79.82 | 76.18 | 4.65 |
| M3 | 75.94 | 65.74 | 73.13 | 74.49 | 75.75 | 74.28 | 68.82 | 73.41 | 72.91 | 71.89 | 72.64 | 3.01 |
| M4 | 83.53 | 81.44 | 83.67 | 88.50 | 84.72 | 82.08 | 72.35 | 85.06 | 79.98 | 84.48 | 82.58 | 4.06 |
| M5 | 79.75 | 77.30 | 70.30 | 74.55 | 78.77 | 79.00 | 80.72 | 77.65 | 76.18 | 71.66 | 76.59 | 3.28 |

***Table S2: Raw data underlying Figure 5.*** *For the six ANN architectures detailed in Table 2 (first column), the accuracies of ten training runs with N*_cal_*=300 are indicated, as well as their mean and standard deviation (std. dev.).*
